# Construction of a doxycycline inducible adipogenic lentiviral expression system

**DOI:** 10.1016/j.plasmid.2012.10.001

**Published:** 2013-01

**Authors:** Q. Liu, P.J. Hill, A. Karamitri, K.J.P. Ryan, H.Y. Chen, M.A. Lomax

**Affiliations:** School of Biosciences, University of Nottingham, Sutton Bonington Campus, Loughborough, Leicestershire LE12 5RD, United Kingdom

**Keywords:** Tetracycline inducible expression, Lentiviral plasmid, Adipose-specific promoter, Adipocyte

## Abstract

To provide a tool for research on regulating adipocyte differentiation, tetracycline inducible (Tet on) lentiviral expression vectors under the control of an adipose-specific promoter were constructed. The lowest basal expression in the absence of doxycycline and most efficient dose-dependent, doxycycline-induced transient overexpression was observed using vectors constructed with a combination of Tetracycline Responsive Element (TRE) and reverse tetracycline-controlled TransActivator advanced (rtTAadv), transfected in white (3T3-L1) and brown (HIB-1B) preadipocytes cell lines. The results demonstrate that doxycycline adipogenic inducible expression can be achieved using a pLenti TRE / rtTA adv under the control of the truncated aP2 promoter in HIB-1B preadipocytes.

## Highlights

► We constructed a lentiviral Tet On overexpression plasmid controlled by aP2 promoter. ► The combination of TRE + rtTAadv in vectors resulted in the lowest basal expression. ► TRE + rtTAadv also gave the best inducibility by Dox in HIB-1B and 3T3-L1 cell lines. ► A truncated aP2 promoter conferred adipogenic expression in HIB-1B cells.

## 1 Introduction

Adipose tissue is found in two forms, energy-storing white adipose tissue (WAT), and energy expending brown adipose tissue (BAT). It has been proposed that the balance between these two adipose tissue types may contribute to the development of obesity (Klingenberg, 1999; Nedergaard et al., 2001). Adipogenesis is controlled by a complicated regulatory network involving the time-dependent expression of a series of transcription factors, co-regulators and signaling pathways (Morrison and Farmer, 1999; Rosen and Spiegelman, 2000; Yeh et al., 1995). Induction of differentiation in white preadipocytes is orchestrated by the temporal expression of C/EBPβ, C/EBPα and PPARγ while brown adipocytes are thought to emanate from a myogenic progenitor as a result of expression of PRDM16 and C/EBPβ (Kajimura et al., 2009, 2008; Seale et al., 2008). The purpose of the experimental work reported here was to construct an adipose tissue specific promoter (aP2) driving a tetracycline inducible (Tet on) lentiviral expression backbone, so that the vectors can be used in the future to produce lentiviruses for the construction of transgenic cell lines.

Tetracycline has been employed to regulate gene expression by binding to the Tet Operator (tetO) within the tetracycline response element (TRE) to either switch off expression (tet-Off) or switch on expression (tet-On). The Tet On expression system has two essential components, the TRE and the reverse tetracycline-controlled transactivator (rtTA), both of which have evolving design and sequences. The original TRE-based promoter (Gossen and Bujard, 1992) confers inducible expression in the presence of doxycycline but has a relatively high background transcription (“leakiness”). The improved TRE promoter, TRE tight (Clontech) has been suggested to show a greater response to doxycycline coupled with extremely low basal transcriptional activity by redesign of the 7 tetO sequences that make up the TRE and the removal of the potential binding sites of endogenous transcription factors (Clontechniques, 2003). The Tet On transactivator rtTA also has been improved (rtTA advanced also known as rtTA2^S^-M2) by utilizing human codon preferences, removing cryptic splice sites from the mRNA sequences to increase its sensitivity to doxycycline and significantly diminish residual binding to TREs in the absence of doxycycline (Urlinger et al., 2000). Therefore there are two versions of TREs and two versions of rtTAs, thus four different combinations were tested in order to determine which backbone gave the best doxycycline inducible expression. Previous studies employing adipose-specific (adipogenic) expression vectors have used the adipose tissue specific promoter aP2 promoter (P_aP2_). However, the size (5.6 kb) of the aP2 promoter exceeds the maximal insert size (4–5 kb in total) of pLenti6 destination vector (Invitrogen), so a 1.2 kb truncated aP2 promoter (P′_aP2_) containing only the fat-specific enhancer and the proximal promoter of the original aP2 promoter (Graves et al., 1992), was used to generate the vectors for lentivirus production.

## 2 Materials and methods

### 2.1 Cell culture, transfection and luciferase assay

3T3-L1 cells (ATCC) and HIB-1B cells (kindly provided by B. Spiegelman) were maintained in Dulbecco’s modified Eagle’s medium (DMEM) with 10% (v/v) fetal bovine serum (Invitrogen) and 1% (v/v) sodium pyruvate in 5% CO_2_. Differentiated 3T3-L1 cells (dif 3T3-L1) were obtained by inducing the cells (2 days post-confluence) by 500 μM 3-isobutyl-1-methylxanthine (IBMX), 250nM dexmethasone (Dex) and 167 nM insulin in growth medium for 48 h and then fed with fresh growth medium supplemented with 167 nM insulin for 8 days (Karamanlidis et al., 2007).

For luciferase assay, HIB-1B and 3T3-L1 cells were passaged and plated in 96- and 24- well plates, respectively, for 24 h before transfection at 70–80% confluence. For selecting the best inducible lentiviral expression backbone, the constitutive or inducible LucGFP lentiviral expression vector (100 ng/well in 96-well plate and 300 ng/well for 24-well plate) was transfected to above cells with FugeneHD® (Roche) according to the manufacturer’s instructions. Twenty-four hour after transfection, 1 μg/ml doxycycline was added to the cells to induce LucGFP expression, and treated for 24 h. For selecting the best adipogenic lentiviral expression backbone, the P_aP2_ or P′_aP2_ driving inducible LucGFP lentiviral expression vectors were transfected into the plated cells with or without the co-overexpression of C/EBPβ and PPARγ (50 ng/well for 24-well plate) using FugeneHD®. Twenty-four hour post transfection, 10 μM rosiglitazone and/or 1 μg/ml doxycycline (Dox) was added to the cells and treated for 24 h. If needed, 10 μM forskolin was added to the cells 36 h post-transfection and treated for 12 h. The cells were then assayed by Firefly Luciferase Assay System (Promega). Cotransfection with a Renilla control plasmid was omitted from this series of experiments as each vector was compared with its own no doxycycline control, the plasmid vectors were of similar sizes and no variations in transfection efficiency were predicted, as supported by the relatively small standard error of the mean values.

### 2.2 Plasmids used in this study

pLenti6/V5 lentiviral expression backbone was from Invitrogen. The templates for cloning TRE and TRE tight, pL3-TRE-LucGFP-2L (Plasmid 11685) and pTRE-Tight miR-1 (Plasmid 14896), respectively, and the template for cloning P_aP2_ and P′_aP2_, pBS-aP2 (Plasmid 11424) all came from Addgene. The template for cloning rtTA, pTet On was from Clontech and the template for cloning rtTA adv, PB-CA-rtTA advance was from Addgene. The overexpression vectors pcDNA3.1-C/EBPβ (Plasmid 12557) and pcDNA-PPARγ (Plasmid 8895) were also from Addgene.

### 2.3 Construction of Tet on and fat-specific Tet on Luciferase-GFP (LucGFP) lentiviral expression vectors

**Fig. 1:**
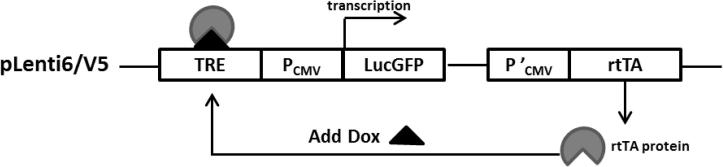
Schematic working process of Tet On lentiviral expression vectors. The TRE is located upstream of the CMV promoter (P_CMV_) driving the target gene LucGFP which is silent in the absence of activation by the reverse tetracycline-controlled transactivator (rtTA). The rtTA is transcribed and translated and binds to the TRE but only activates transcription of the target gene LucGFP in the presence of Dox.

The 3-fragment MultiSite Gateway® (Invitrogen) cloning system was used to create Luciferase-GFP (LucGFP) fusion protein lentiviral expression vectors, so the reporter gene LucGFP, transactivator and the promoter driving transactivator could be simultaneously cloned into the modified Tet On lentiviral destination vector. The constructed vectors were identified by restriction digest and vector sequencing. The Tet On vectors worked as illustrated in Fig. 1.

#### 2.3.1 Construction of Tet on lentiviral destination vectors

The tetracycline response element (TRE) and the improved version TRE tight were amplified by PCR from pL3-TRE-LucGFP-2L and pTRE-Tight miR-1 respectively, and *Cla*I and *Spe*I restriction sites were added to the 5′ and 3′ end of the PCR products (primer sequences in Table 1). The digested (*Cla*I + *Spe*I) PCR products and pLenti6/V5 plasmid were then ligated to create new Tet on lentiviral destination vectors, pLenti TRE and pLenti TRE tight.

#### 2.3.2 Construction of short aP2 promoter (P′_aP2_) template vector

The fat-specific enhancer and the proximal promoter of aP2 were amplified by PCR from pBS-aP2 vector (primer sequences in Table 1), and *Not*I restriction site was added to both 5′ and 3′ ends of the PCR product, which was digested with *Not*I and self-circulated to generate the pBS-aP2′ template vector for amplifying P′_aP2_.

#### 2.3.3 Entry clones for 3-fragment MultiSite Gateway® recombination

Entry clones of LucGFP, rtTA or rtTA adv and P_CMV_, P_aP2_ or P′_aP2_ were created by BP reactions according to the protocol from Invitrogen. PCR primers for amplifying each target sequence and adding recombination sites (att sites) were listed in Table 1.

#### 2.3.4 Construction of lentiviral expression vectors

LR reactions were performed according to the protocol from Invitrogen, to construct the lentiviral expression vectors.

**Fig. 2:**
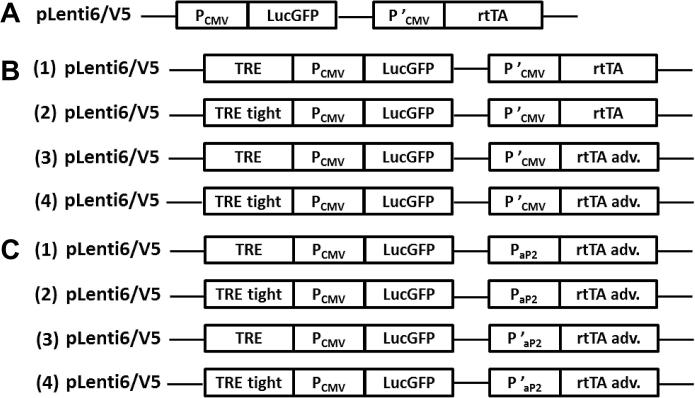
Schematic of the lentiviral LucGFP expression vectors. (A) The constitutive pLenti LucGFP expression vector was created by 3-fragment MultiSite Gateway® cloning system (LR reaction) using pLenti6/V5 and entry clones of LucGFP, P′_CMV_ and rtTA. (B) The TRE (1, 3) or TRE tight (2, 4) element was inserted upstream of the CMV promoter (P_CMV_) in the pLenti6/V5 vector using BamHI and XhoI. The LucGFP, P′_CMV_ and rtTA or rtTA adv entry clones were cloned into the modified backbones by LR reaction. (C) The full length (P_aP2_) or truncated (P′_aP2_) aP2 promoter was inserted upstream of the transactivator rtTA adv element in either the TRE or TRE tight P_CMV_ / LucGFP pLenti backbone by the 3-fragment MultiSite Gateway® cloning system.

Entry clones of LucGFP, P_CMV_ and rtTA and the original pLenti6/V5 destination vector were used to generate constitutive LucGFP lentiviral expression vector, pLenti-LucGFP (Fig. 2A). Entry clones of LucGFP, P_CMV_ and rtTA or rtTA adv and the Tet on lentiviral destination vectors (pLenti TRE or pLenti TRE tight) were used to generate Tet on LucGFP lentiviral expression vectors (Fig. 2B). Entry clones of LucGFP, P_aP2_ or P′_aP2_ and rtTA adv and the destination vector pLenti TRE or pLenti TRE tight were used to generate adipocyte-specific inducible lentiviral expression vectors (Fig. 5).

### 2.4 Quantitative real time PCR

Total RNA was isolated using Trizol Reagent (Invitrogen) according to the manufacturer’s instructions. A 0.2 μg sample of total RNA was employed for cDNA synthesis using the Omniscript® Reverse Transcriptase Kit (Qiagen), with Random Primer (Promega). For amplification of aP2 cDNA, the primer sequences were 5′-AGCATCATAACCCTAGATGG-3′ (forward) and 5′-GAAGTCACGCCTTTCATAAC-3′ (reverse), and the expected size of the PCR product was 128 bp. The cDNA was amplified in a 15 μl reaction with LightCycler® 480 DNA SYBR Green I Master Mix (Roche). LightCycler® 480 Instrument was used to perform the PCR reactions with the following temperature profile: an initial denaturation temperature at 95 °C for 15 min, 35 cycles of denaturation (20 s at 95 °C), annealing (20 s at 58 °C) and extension (20 s at 72 °C). The data acquisition was performed during the extension period and a melting curve was acquired between 72 °C and 95 °C to check for primer dimers or other non-specific amplicons. The specificity of PCR products were also checked by agarose gel electrophoresis. The results were normalized against 36B4 housekeeping gene expression and calculated using Δ (ΔCt) method.

### 2.5 Statistical analysis

Data in Figures is presented as average ± SEM from 2 or 3 independent replicate experiments with duplicate or triplicate wells in each experiment. Effects of treatments were determined by performing Student’s *t*-test or Analysis of Variance (ANOVA) as indicated in individual Figure legend. Significance was accepted if *P* < 0.05. All statistical analyses were performed on SPSS statistical package version 16 (SPSS Inc. Chicago, IL, USA).

## 3 Results and discussion

### 3.1 Comparison of inducible lentiviral LucGFP expression vectors

**Fig. 3:**
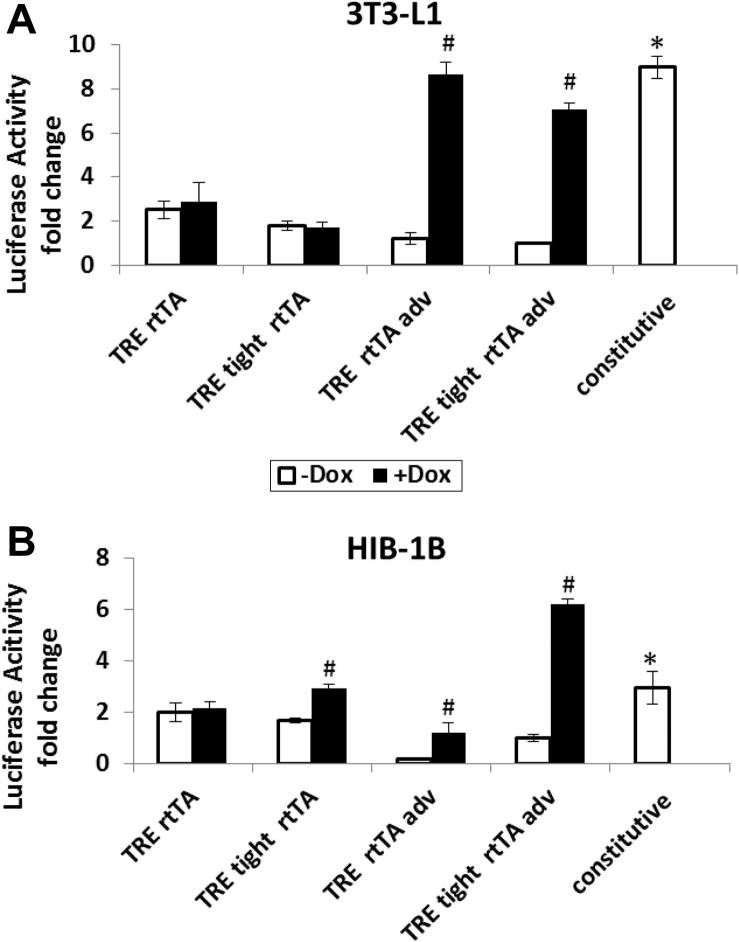
Comparison of different Tet on LucGFP vectors (CMV driven) by luciferase assay in 3T3-L1 (A) and HIB-1B (B) preadipocytes. HIB-1B and 3T3-L1 preadipocytes were transiently transfected with different Tet On CMV-LucGFP vectors, the empty vector pLenti TRE (negative control) or the constitutive vector pLenti-LucGFP (CMV, positive control). Cells were given 1 μg/ml doxycycline (Dox) 24 h post-transfection and cultured for 24 h. Luciferase activity was expressed as fold change relative to the value from the cells transfected with pLenti TRE tight-LucGFP-rtTA adv vector without doxycycline induction. Results represent mean ± S.E.M from 2 independent replicate experiments performed in triplicate wells. Student’s *t*-test was used to analyze the data. ^∗^*P* < 0.05 with respect to basal TRE rtTA; #*P* < 0.05 with respect to the same vector without Dox treatment.

The four Tet On inducible pLenti LucGFP expression vectors constructed using different versions of TRE and rtTA (Fig. 2), were transiently transfected into 3T3-L1 and HIB-1B preadipocytes on separate occasions. Cells were treated with 1 μg/ml doxycycline (Dox) 24 post-transfection and then cultured for 24 h before luciferase assay to compare the response to Dox above baseline. The combinations of TRE and rtTA, and TRE tight and rtTA, resulted in poor responses to Dox above baseline, in both 3T3-L1 and HIB-1B cells (Fig. 3A). The vectors constructed using TRE and rtTA advance, and TRE tight rtTA advance exhibited low basal activity in the absence of Dox and the induced activity in the presence of Dox (5.5 and 6-fold induced, respectively, *P* < 0.05) were comparable with the constitutive (non-inducible) vector in 3T3-L1 cells (Fig. 3A). In HIB-1B cells (Fig. 3B), the vector combining TRE with rtTA advance had a low basal activity and better Dox induciblity (7.7-fold induced, *P* < 0.05), but the absolute value of induced luciferase activity was only half of the constitutive vector. Similar to the result in 3T3-L1, pLentiTRE tight LucGFP rtTA advance in HIB-1B cells also had relative low leakiness and good inducibility (6.2-fold induced, *P* < 0.05), and the induced luciferase activity was nearly double that of the constitutive vector value. The results demonstrated clearly that the improved version of transactivator rtTA adv could significantly decrease the basal expression of the vectors in the absence of Dox, compared with the original transactivator rtTA. In terms of the absolute expression level in presence of Dox, the combination of TRE with rtTA adv performed slightly better than the combination of TRE tight with rtTA adv in 3T3-L1 cells, but in HIB-1B cells the Dox induced expression level of TRE + rtTA adv was much lower than that of TRE tight + rtTA adv, which might reflect the various sensitivity of different cell lines to the elements involved in the Tet on response.

**Fig. 4:**
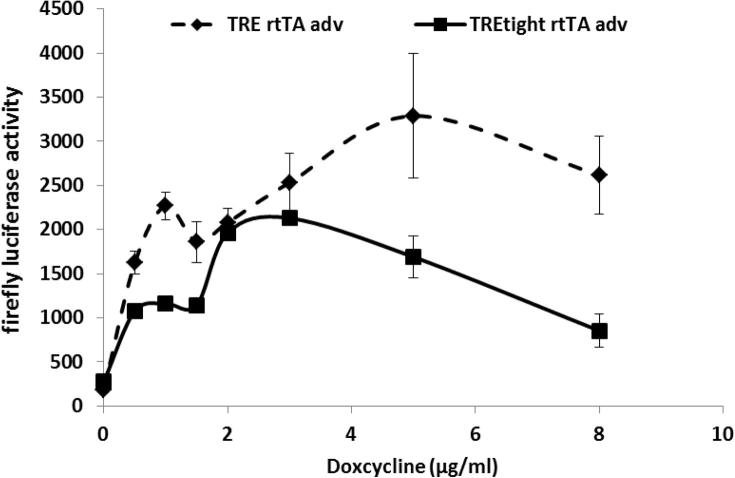
The Tet on LucGFP vectors responded to doxycycline in a dose-dependent manner. 3T3-L1 preadipocytes were transfected with either TRE LucGFP rtTA adv or TRE tight LucGFP rtTA adv vector. Doxycycline was added into the cells 24 h post-transfection at the concentration indicated in the graph. Firefly luciferase activity was measured 24 h after the addition of doxycycline. The error bars represent S.E.M from 2 independent replicate experiments performed in triplicate wells.

Expression of Tet on vectors can be induced by Dox in a dose-dependent manner (Tang et al., 2009), so dose response experiments using the two most responsive vectors (TRE / rtTA adv and TRE tight / rtTA adv) were performed in the 3T3-L1 cell line (Fig. 4). The results demonstrated that the TRE LucGFP rtTA adv vector was more sensitive to low concentrations of doxycycline than TRE tight LucGFP rtTA adv vector, since the luciferase activity was upregulated 12 and 5-fold, respectively, at 1 μg/ml Dox. Above 1 μg/ml Dox, the response of both vectors was more variable and the induction using 8 μg/ml of Dox was reduced, compared to that using 2–3 μg/ml suggesting possible toxic effects of higher concentrations of Dox in this cell line (Fig. 4).

### 3.2 Comparison of aP2 inducible lentiviral LucGFP expression vectors

**Fig. 5:**
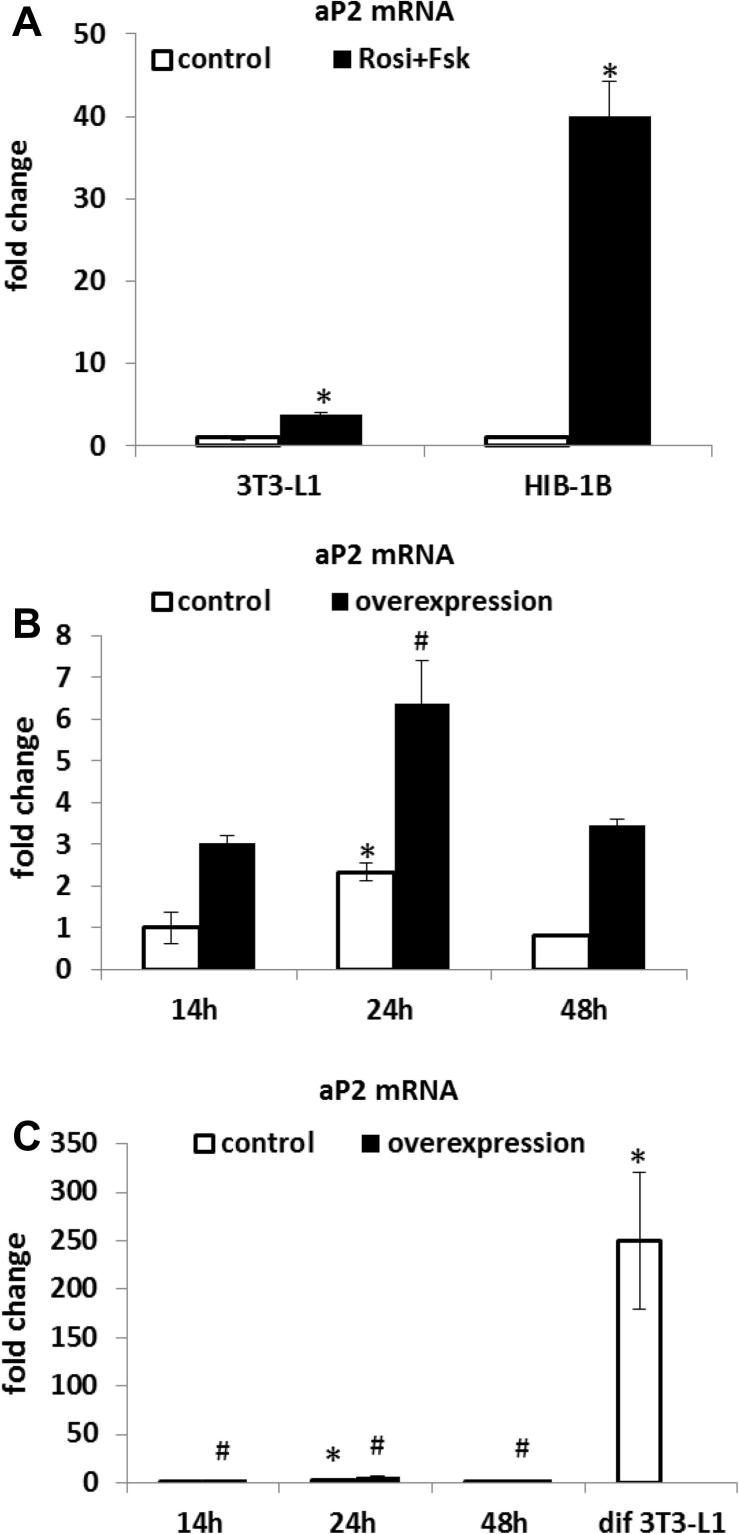
Adipogenic stimulation of aP2 expression by acute treatment of 3T3-L1 and HIB-1B cells. 10 μM rosiglitazone (Rosi) were added to confluent HIB-1B and 3T3-L1 (A) cells and 10 μM forskolin (Fsk) was added to the culture 12 h later. RNA was extracted from the cells 12 h after adding forskolin and aP2 mRNA expression levels in treated 3T3-L1 and HIB-1B cells was determined by quantitative real time PCR and normalized against 36B4 housekeeping gene expression. (B) 3T3-L1 preadipocytes were transfected with C/EBPβ and PPARγ by FugeneHD®. 10 μM rosiglitazone (Rosi) were added into the cells 24 h post-transfection and RNA was extracted from the cells 24 h after adding rosiglitazone. (C) The differentiated 3T3-L1 cells (dif 3T3-L1) were derived from a standard differentiated protocol outlined in the Methods section. The level of aP2 mRNA in treated cells was determined by quantitative real time PCR and normalized against 18s housekeeping gene expression, relative to the value from the 14 h pcDNA transfected cells. Each bar represents the mean ± S.E.M from 2 independent replicate experiments performed in duplicate wells. (A) ^∗^*P* < 0.05 by Student’s *t*-test with respect to controls. (B) (C) Student’s *t*-test: ^∗^*P* < 0.05 caused by time of Rosi treatment or differentiation with respect to the control at 14 h; #*P* < 0.05 caused by overexpression with respect to the control at the same time point.

In order to evaluate vectors employing different forms of the adipogenic-specific aP2 promoter it was first necessary to establish the conditions capable of inducing expression of aP2 within 48 h, in transiently transfected confluent preadipocytes. Preliminary experiments were performed to measure changes in aP2 mRNA when adipocytes were stimulated by adding a cocktail of two drugs previously shown to initiate adipogenesis namely 10 μM rosiglitazone (a PPARγ agonist) (Lehrke and Lazar, 2005) and 10 μM forskolin (an activator of the β-adrenergic signaling downstream target adenylyl cyclase) (Yarwood et al., 1995). aP2 expression in HIB-1B cells treated for 24 h with rosiglitazone and forskolin was increased by 65-fold (*P* < 0.001) compared with the controls (Fig. 5A), but the same experiment in 3T3-L1 cells only increased aP2 expression by around 4-fold (*P* < 0.05) (Fig. 5A). Since expression of C/EBPβ and PPARγ both play critical roles in the early adipogenic programme in 3T3-L1 cells (Lane et al., 1999; Tontonoz et al., 1994), a further experiment was performed to examine the induction of aP2 gene expression by transiently overexpressing C/EBPβ and PPARγ in 3T3-L1 cells which were treated with 10 μM rosiglitazone over a 48 h period. aP2 gene expression was stimulated 3–4-fold (*P* < 0.05) by co-overexpression of C/EBPβ and PPARγ compared to transfected control cells (Fig. 5B), with this response being maximal at 24 h after the addition of rosiglitazone. This level of aP2 induction was more than a hundred fold less than that observed in 3T3-L1 cells 10 days after inducing differentiation using the standard differentiation protocol (IBMX, Dex and insulin) as described in the Methods section (Karamanlidis et al., 2007), indicating that the cells were early in the differentiation programme (Fig. 5C).

**Fig. 6:**
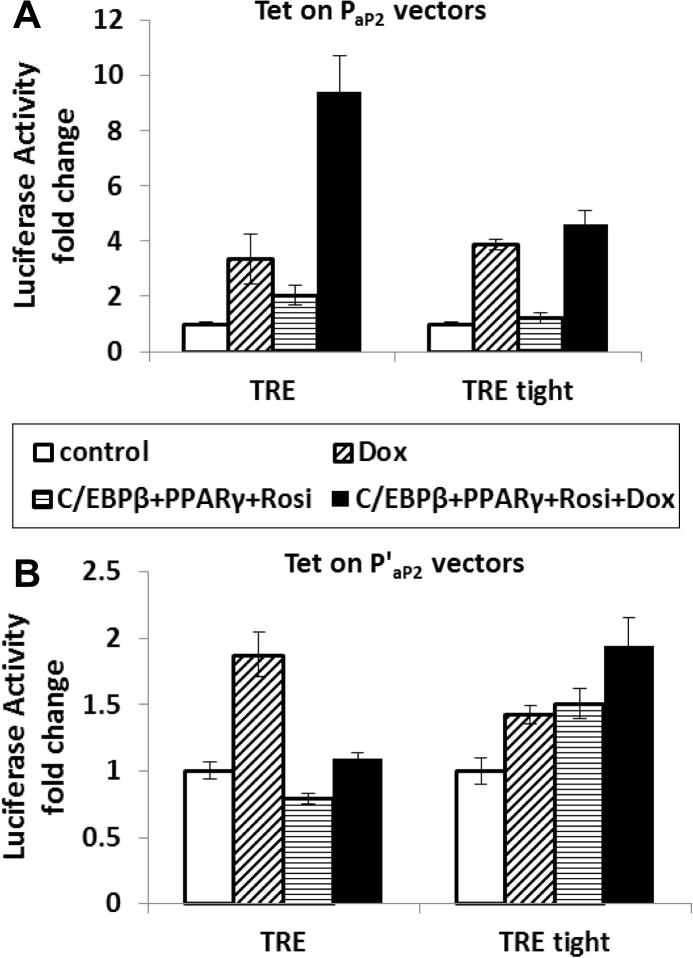
Activity of adipogenic and doxycycline inducible lentiviral LucGFP expression vectors in 3T3-L1. The inducible LucGFP lentiviral vectors with full length aP2 promoter (Tet On P_aP2_ vectors, A) or with truncated aP2 promoter (Tet on P′_aP2_ vectors, B) were co-overexpressed with C/EBPβ and PPARγ into 3T3-L1 preadipocytes. 10 μM rosiglitazone (Rosi) and 1 μg/ml doxycycline (Dox) were added to the cells 24 h post-transfection as indicated and treated for 24 h before luciferase assay. Firefly luciferase activity was interpreted relative to the control value (no co-overexpression, no drug treatments) of each group. Each bar represents the mean ± S.E.M from 2 independent replicate experiments performed in triplicate wells. Data was analyzed by two-way ANOVA for each lentiviral vector to examine the effects of Dox treatment and application of adipogenic conditions (C/EBPβ + PPARγ co-expression + Rosi treatment). (A) TRE P_aP2_ (left): Dox (*P* < 0.001) and adipogenic condition (*P* < 0.001) both significantly increased P_aP2_ activity and there was no interaction (*P* = 0.18) between the two treatments; TRE tight P_aP2_ (right): Dox (*P* < 0.001) significantly increased P_aP2_ activity while adipogenic conditions had no effect (*P* = 0.31) and there was no interaction (*P* = 0.091). (B) Neither Dox nor adipogenic condition treatment had significant effects on P′_aP2_ activity.

**Fig. 7:**
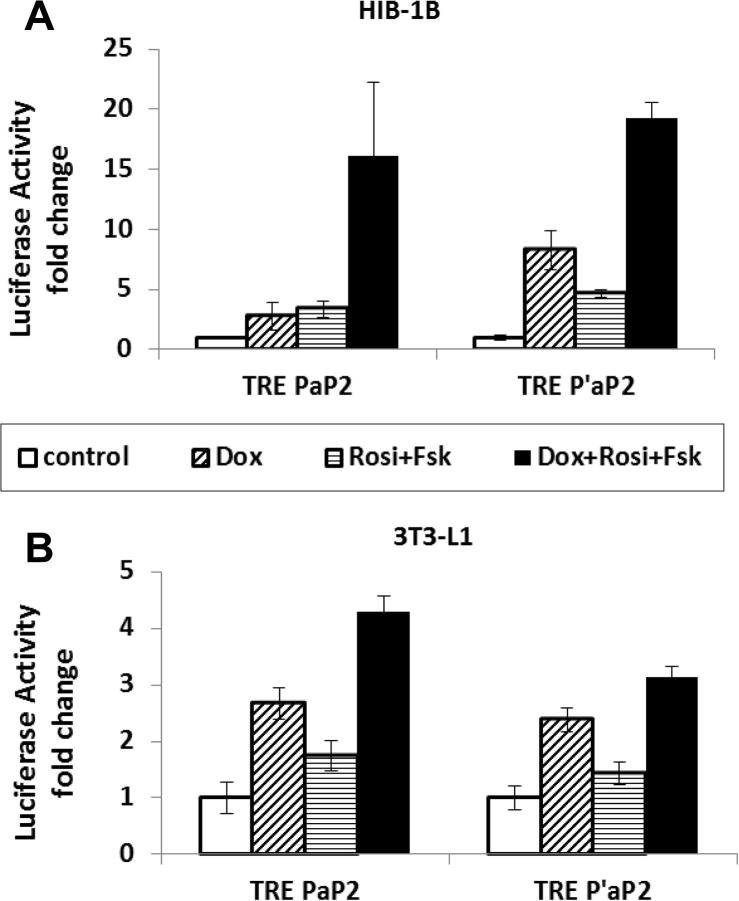
Activity of adipogenic and doxycycline inducible lentiviral LucGFP expression vectors in 3T3-L1 and HIB-1B preadipocytes. The two fat specific expression vectors, pLenti TRE-LucGFP P_aP2_ rtTA adv and pLenti TRE-LucGFP P′_aP2_ rtTA adv were transiently expressed into HIB-1B (A) or 3T3-L1 (B) preadipocytes. 10 μM Rosiglitazone (Rosi) and 10 μM forskolin (Fsk), as indicated, were added to the cells 24 and 36 h post-transfection respectively. 1 μg/ml doxycycline (Dox) was added to the cells 24 h post-transfection to induce the expression of the Tet on vectors. Luciferase assay was carried out 48 h after transfection and firefly luciferase activity was interpreted relative to the control value (no Rosi, no Fsk, and no Dox) of each group. Each bar represents the mean ± S.E.M from 2 independent replicate experiments performed in triplicate wells. Data was analyzed by two-way ANOVA with each lentiviral vector. (A) left: no interaction between Dox and Fsk + Rosi treatments (*P* = 0.111) but Dox (*P* < 0.001) and Fsk + Rosi (*P* < 0.001) both independently increased P_aP2_ activity; right: no interaction between Dox and Fsk + Rosi treatments (*P* = 0.459) while Dox (*P* < 0.001) and Fsk + Rosi (*P* = 0.008) both independently increased P′_aP2_ activity. (B) No interaction between Dox and Fsk + Rosi treatments (*P* > 0.05) and Dox and fsk + Rosi independently increased both P_aP2_ and P′_aP2_ activity (*P* < 0.001).

The four vectors employing the two successful combinations of Tet on elements described above (TRE / rtTA adv or TRE tight / rtTA adv) in which the rtTA adv was controlled either by the full length aP2 promoter (P_aP2_) or a truncated promoter (P′_ap2_) constituting the enhancer and proximal promoter elements (Fig. 2), were transiently transfected into 3T3-L1 cells. Relative to the cells without any induction (control cells), addition of Dox stimulated a 4-fold increase in promoter activity from the P_aP2_ and TRE / rtTA adv combination vector (Fig. 6A; *P* < 0.05). This effect was further accentuated in 3T3-L1 cells treated to adipogenic induction (co-overexpression of C/EBPβ and PPARγ plus rosiglitazone treatment), with Dox treatment inducing a 9-fold increase in promoter activity relative to control cells (*P* < 0.001). The vector combining P_aP2_ and TRE tight / rtTA adv was induced 4-fold by Dox (*P* < 0.001; Fig. 6A) however adipogenic induction treatment had no further stimulatory effect. In the vectors constructed with truncated aP2 promoter (P′_aP2_), neither the TRE-rtTA adv nor TRE tight rtTA adv combination were able to respond to doxycycline or adipogenic treatment to the extent observed with the full length aP2 promoter (Fig. 6B). When the aP2 promoter was induced by combined induction with rosiglitazone and forskolin, there was a 15-fold induction of luciferase activity in HIB-1B cells transiently transfected with TRE-rtTA adv under the control of either P_aP2_, or P′_aP2_, in response to Dox (Fig. 7A; *P* < 0.001). The same experiments in 3T3-L1 cells showed lower responses (3–4-fold) to Dox and smaller responses to adipogenic induction using rosiglitazone and forskolin (Fig. 7B).

### 3.3 Discussion

This study demonstrates clear differences in basal and Dox induced expression in vectors constructed using different combinations of tetracycline response elements (TRE or TRE tight) with reverse tetracycline-controlled transactivators (rtTA or rtTA adv) transiently transfected into white (3T3-L1) and brown (HIB-1B) preadipocytes. The most efficient Dox inducibility with the lowest basal expression was achieved with an expression construct combining TRE and rtTA adv, (i.e. pLentiTRE-LucGFP rtTA adv) in both cell types. There are two potential reasons responsible for differences in basal expression, 1) the residual binding of rtTA to tetO sequence of TRE in the absence of inducer ligand doxycycline (Baron and Bujard, 2000) and 2) the basal transcriptional activity of the tetracycline responsive promoter even without binding to rtTA. Replacing the TRE with TRE tight (the latter was supposed to be a more tightly controlled tetracycline response element), did not improve the inducibility but the basal activity was slightly reduced (Fig. 3), indicating that the residual binding of rtTA to tetO sequence is the primary reason contributing to the “leakiness” of the vectors. Replacing the original rtTA with rtTA adv, which contains specific mutations that both increase its sensitivity to doxycycline and significantly diminish residual binding to tetO sequence in the absence of inducer ligand (Urlinger et al., 2000), greatly improved the inducibility of the Tet on vectors with lower basal activity (50% decreased) and produced a much higher induced expression (7–8-fold)(Fig. 3). (Urlinger et al., 2000) reported that rtTA gave poor responses in some cell lines with a relatively low sensitivity toward Dox, therefore the lack of (or poor) induction with the rtTA vector was not surprising. It was concluded that the most efficient response to Dox with the lowest basal expression was found using a combination of TRE and rtTA adv in both 3T3-L1 and HIB-1B cells.

When the adipogenic promoters were introduced into the inducible expression vectors, the difference between the two inducible backbones was even more obvious. 3T3-L1 cells expressing the full length P_aP2_ driving TRE rtTA advance vector, in the presence of doxycycline and adipogenic induction (overexpression of C/EBPβ, PPARγ in the presence of rosiglitazone), demonstrated a 9-fold increase in luciferase activity compared to unstimulated control cells. In contrast the luciferase activity of P_aP2_ driving TRE tight rtTA adv vector was increased only 3-fold by doxycycline treatment and this response was not affected in cells exposed to the adipogenic conditions (Fig. 6A). This difference demonstrated that some specific regulatory sequences in the TRE tight element might influence the activity of P_aP2_.

Rosiglitazone and forskolin treatment was able to successfully induce expression 15-fold when the TRE / rtTA advance vector was placed under the control of either the full length P_aP2_ or truncated P′_aP2_, transfected into HIB-1B cells and stimulated with Dox (Fig. 7A). However in 3T3-L1 cells, the truncated P′_aP2_ TRE / rtTA advance vector construct was not significantly increased by adipogenic stimulation (Figs. 6 and 7B). It is well known that differentiation of 3T3-L1 cells requires a critical early phase of mitotic clonal expansion (MCE), when the confluent growth arrested cells re-enter cell cycle and prepare for the differentiation for 48 h (Tang et al., 2003). It has been demonstrated that the MCE is a prerequisite for differentiation of 3T3-L1 preadipocytes to adipocytes, but the differentiation of HIB-1B preadipocytes does not require this phase. This difference could possibly explain why the same adipogenic induction conditions worked well in HIB-1B but had poor effect in 3T3-L1 preadipocytes since the MCE was omitted as it would not have allowed measurement of the reporter expression from the transiently transfected vectors. A further limitation of the study was that the adipose-specific nature of the constructs was not tested in other cell lines.

### 3.4 Conclusion

We have constructed an inducible LucGFP expression vector comprising of LucGFP regulated by TRE-P_CMV_ and induced by rtTA regulated by the truncated aP2 promoter. This expression construct gave low basal, high Dox inducibility under adipogenic conditions when transiently transfected into HIB-1B brown preadipocytes.

## Figures and Tables

**Table 1:** Primer sequences for cloning.

Target		Primer sequence (5′-3′)	Flanking inserts	Template
TRE	F	acgaagttatATCGATgaaccccttcc	*ClaI* site	pL3-TRE-LucGFP-2L
	R	ccaagcttagaACTAGTggatcggtcccggtgtcttc	*SpeI* site	
TRE tight	F	ccccgggATCGATggccctttcgtcttcactcgag	*ClaI* site	pTRE-Tight miR-1
	R	ccccgggACTAGTgcgatctgacggttcactaaac	*SpeI* site	
pBS-aP2′	F	cccggGCGGCCGCccaacccaaaccaaacaaagccaaac	*NotI* site	pBS-aP2
	R	cccggGCGGCCGCggttctgactcctggcctgaacttc	*NotI* site	
LucGFP	F	*GGGG ACA AGT TTG TAC AAA AAA GCA GGC T*ccaccatggaagacgccaaaaac	attB1 site	pL3-TRE-LucGFP-2L
	R	*GGGG AC AAC TTT GTA TAG AAA AGT TGG G*tgagaagagggacagctatgac	attB4 site	
P_CMV_	F	*GGGG ACA ACT TTT CTA TAC AAA GTT G*tattggctcatgtccaacattaccgcc	attB4r site	pL3-TRE-LucGFP-2L
	R	*GGGG AC AAC TTT ATT ATA CAA AGT TGT*gagctctgcttatatagacctcc	attB3r site	
P_aP2_	F	*GGGG ACA ACT TTT CTA TAC AAA GTT G*atatcgaattcccagcaggaatcaggtagc	attB4r site	pBS-aP2
	R	*GGGG AC AAC TTT ATT ATA CAA AGT T GT*ctgcagcacaggagggtgctatgagcc	attB3r site	
P′_aP2_	F	*GGGG ACA ACT TTT CTA TAC AAA GTT G*atatcgaattcccagcaggaatcaggtagc	attB4r site	pBS-aP2′
	R	*GGGG AC AAC TTT ATT ATA CAA AGT T GT*ctgcagcacaggagggtgctatgagcc	attB3r site	
rtTA	F	*GGGG ACA ACT TTG TAT AAT AAA GTT G*atccagcctccgcggccccg	attB2 site	pTet-on
	R	*GGGG AC CAC TTT GTA CAA GAA AGC TGG GTA*gcttggtcgagctgatacttcccgtcc	attB3 site	
rtTA adv	F	*GGGG ACA ACT TTG TAT AAT AAA GTT G*gcaggcttcaccatgtctagactggac	attB2 site	PB-CA-rtTA advance
	R	*GGGG AC CAC TTT GTA CAA GAA AGC TGG GTA*ggtcgagggatcttcataagagaagaggg	attB3 site	
